# Learner-Controlled Self-Observation is Advantageous for Motor Skill Acquisition

**DOI:** 10.3389/fpsyg.2012.00556

**Published:** 2013-01-17

**Authors:** Diane M. Ste-Marie, Kelly A. Vertes, Barbi Law, Amanda M. Rymal

**Affiliations:** ^1^School of Human Kinetics, University of OttawaOttawa, ON, Canada; ^2^School of Physical and Health Education, Nipissing UniversityNorth Bay, ON, Canada; ^3^Department of Kinesiology, San Francisco State UniversitySan Francisco, CA, USA

**Keywords:** self-control, observational learning, self-efficacy, intrinsic motivation

## Abstract

There were two main objectives of this research. First, we wanted to examine whether video feedback of the self (self-observation) was more effective for motor skill learning when the choice to view the video was provided to the learner (learner-controlled, LC) as opposed to an experimenter-controlled (EC) delivery. Secondly, we explored whether there were differences in the self-regulatory processes of self-efficacy and intrinsic motivation, as well as perceived choice between the LC and EC conditions. Two groups (LC and EC) of children (*M* age of 11.2 years; SD = 1.89) attempted to learn a progression of trampoline skills during a 2-day acquisition phase in which video self-observation was available. The second acquisition day was followed by a no self-observation retention test 1 day later. It was hypothesized that, during retention, the LC group would be more self-efficacious about their ability to progress through the trampoline skills, show greater intrinsic motivation and perceived choice, and go further in skill progression than the EC group. Analysis of the acquisition data showed the LC group had greater increases in self-efficacy as compared to the EC group. Results of the retention test showed that the participants in the LC group obtained higher scores on the intrinsic motivation and perceived choice measures and had higher skill progression scores as compared to the EC group. Regression analysis showed that group assignment and self-efficacy were significant predictors of the physical performance benefits noted in retention. These findings are discussed within Zimmerman’s (2004) self-regulation of learning model.

## Introduction

A number of convergent research areas suggest that allowing individuals to control features of their own learning environment enhances motor learning. As examples, learner-controlled (LC) conditions for receiving knowledge of results (KR; Patterson and Carter, 2010; Hansen et al., 2011; Patterson et al., 2011) or knowledge of performance (KP; Janelle et al., 1997; Wulf et al., 2005) about motor task performance, the scheduling of practice trials (Keetch and Lee, 2007; Wu and Magill, 2011), and physical assistive device use (Wulf and Toole, 1999; Wulf et al., 2001; Hartman, 2007) have been shown to be superior to experimenter-controlled (EC) conditions. While most of the research has been conducted with healthy adults, learning advantages have also been evidenced in populations with movement disorders (Chiviacowsky et al., 2012a,b) and with children controlling their own KR (Chiviacowsky et al., 2008a,b).

To date, however, no research has been conducted to determine whether there are motor learning advantages for children who are provided with control over when to observe themselves on video following task performance; a technique known as self-observation (Dowrick, 1999). Thus, the first objective of our research was to determine whether LC conditions for self-observation enhance motor skill acquisition in children. To do this, we compared motor skill performance in trampoline skills for one group of children who decided themselves when to self-observe on video (LC) against a group of children who were provided with the same self-observation schedule, but following an EC delivery.

Even though there is clear evidence of the benefits associated with learners being empowered to take control of some aspect of their learning environment, there has been little examination of the underlying mechanisms contributing to these benefits (see Chiviacowsky and Wulf, 2005 for an exception). Instead, possible mechanisms are presented as hypotheses that are yet to be tested fully. For example, some researchers have argued that benefits from LC conditions may occur due to increased motivation (Chen et al., 2002; Chiviacowsky and Wulf, 2005; Wulf et al., 2005), or increased meta-cognitive strategies (Patterson and Carter, 2010; Patterson et al., 2011), or that it may lead to more confidence in the learner’s ability to perform the task (Janelle et al., 1997). We agree with these propositions, but rather than examining them as independent questions, we propose that there is value in adopting a more comprehensive theoretical framework to examine these learning advantages and suggest a self-regulation framework, specifically Zimmerman’s (2000) self-regulation of learning model.

Zimmerman’s (2000) self-regulation model was chosen as the theoretical framework for a number of reasons. First, many of the suggested mechanisms for explaining learning advantages obtained from a LC environment fit well within concepts related to self-regulated learning. Second, given that we are using self-observation, it is relevant that Ste-Marie and colleagues (Clark et al., 2006; Rymal et al., 2010; Ste-Marie et al., 2011a,b; Martini et al., 2011) have shown that this self-regulation model aligns well with self-as-a-model research. Third, Zimmerman’s model is situated within Bandura’s (1986) social cognitive perspective that views learning as an interaction of personal, behavioral, and environmental cyclical processes; an often adopted perspective in observational learning research (see McCullagh et al., 2012). Finally, Zimmerman adopts an event approach to self-regulated learning, thus defining self-regulation as occurring within a temporal entity with a marked beginning and end. Consequently, the various self-regulatory processes and beliefs that surround a learning event can be delineated and examined.

Zimmerman (2000) identified three phases of self-regulation; the forethought, performance control, and self-reflection phase. In brief, the forethought phase involves those self-regulatory processes and beliefs that precede one’s actions and enable it to occur, whereas the performance control phase are those that occur during the actual execution of the action. Finally, the self-reflection phase encompasses a number of self-regulatory processes that are engaged in subsequent to the action. These phases act in a cyclical fashion because the information from prior performance is used to make adjustments to the current efforts and so forth. Our current research focus was on self-efficacy and task interest, both situated within the forethought phase of this model, specifically within the self-motivational beliefs component. We are most interested in this phase due to Zimmerman’s (2008) contention that those who engage in high-quality forethought improve their self-regulatory functioning during subsequent phases. Thus, a unique contribution of this work is to test whether LC environments result in higher levels of self-efficacy and greater task interest. We will further elaborate on the two specific processes of interest.

Perceived self-efficacy relates to beliefs in one’s capability to produce certain tasks (Bandura, 1997). This is an important self-regulatory belief because the more competent learners feel about their capabilities; the more likely they are to strive to achieve their goal (Locke and Latham, 1990). Further, a highly self-efficacious person is more likely to persist at acquiring a skill even when having difficulty progressing (Feltz et al., 2008). Our hypothesis is that the LC environment will result in higher levels of self-efficacy. Our rationale ties in with previous findings concerning learners’ preferences to receive feedback following good performance trials (e.g., Chiviacowsky and Wulf, 2002) and the fact that mastery experiences are considered the strongest predictor of self-efficacy (Bandura, 1997). As such, we expected that the children in the LC conditions would view themselves following successful performance attempts (i.e., a mastery experience) and that viewing a good performance of a task could lead a person to feel more confident about their performance on their upcoming trials. In contrast, those viewing the video via EC conditions would not consistently have the viewings coincide with good task performance, and thus not show similar increases in self-efficacy.

Task interest within Zimmerman’s (2000) model identifies the importance associated with performing a task for its intrinsic properties, rather than external ends, and is very much in line with Deci’s (1975) notions of intrinsic motivation. Indeed, Zimmerman refers to Deci’s work when conceptualizing this component of his model, and therefore we measured task interest by examining participants’ intrinsic motivation. Work by Deci and Ryan (2002) has shown clearly that autonomy in one’s decisions increases intrinsic motivation. Given that the LC condition involves the learner choosing when to view (or not view) the self-observation video, we also tested whether children in the LC group would show higher levels of perceived choice and intrinsic motivation as compared to those children who received an EC schedule.

As mentioned, this is the first study to examine whether children will benefit from LC scheduling of self-observation. To be noted, however, is that our experimental protocol was guided from Janelle et al.’s (1997) research that also investigated the advantages of LC self-observation in adults learning to throw. In that research, when the adults chose to watch the video performance of their last trial, they were also informed of the most relevant aspect of the video performance in which to direct their attention (attentional cueing) and then were provided a transitional statement to assist in correcting the movement pattern on the next trial. This LC group was compared to a yoked group (who also received attentional cueing and transitional statements), a summary KP group, and a KRs only group. The results showed that the LC groups’ performance was superior in comparison to all the other groups.

Despite these results with adults, it is possible that children may respond differently to LC self-observation because of the vital role of attention and memory processes in observational learning. Bandura (1977, 1986) described observational learning as operating through four sub-processes; attention, memory, behavior reproduction, and motivation. Within this cognitive framework, a learner must first attend to the relevant information within the demonstration provided; then translate this relevant information through memory processes into a cognitive representation. Indeed, Yando et al. (1978) asserted that an observers’ cognitive-developmental level was a critical factor influencing modeling benefits due to attention span and memory capacity. Given the developmental differences in youth for various information processing abilities, such as rehearsal strategies (Gallagher, 1984; Gallagher and Thomas, 1984) and other memory control processes (Gallagher, 1982; Gallagher and Thomas, 1986), research with children on LC self-observation is warranted.

One measure that may provide insight into the children’s use of information during the acquisition phase is the scheduling of the feedback requested. Janelle et al. (1997) reported that adults only requested feedback for 11.5% of the trials and the relative frequency of the feedback showed a natural fading schedule; i.e., there were greater demands for feedback in the early trials versus those at the end of acquisition. It is possible that children will ask for a greater percentage of feedback trials as compared to adults to compensate for their diminished cognitive processing capacity.

In sum, our research involved children learning trampoline skills under one of two self-observation conditions. In the LC group, children were able to decide after each trial whether they wanted to view their previous performance attempt on video. Children in the EC group were provided with the same self-observation schedule, however, the schedule was determined by yoking it to a counterpart from the LC group. Four specific hypotheses relating to each of the measures were tested using multiple analyses of variance and independent sample *t*-tests wherein main effects were expected at the group level during retention. Basically, we hypothesized that the LC group would (1) show superior physical performance of the trampoline skills, (2) higher levels of self-efficacy, (3) more intrinsic motivation, and (4) greater perceived choice as compared to the EC group. We also expected that children would request feedback at greater frequencies than that seen in similar research with adults (i.e., Janelle et al., 1997). Finally, a hierarchical regression was performed on the retention data with the expectation that the physical performance scores in retention could be predicted by group assignment and the psychological variables measured.

## Materials and Methods

### Participants

Sixty male and female (*M* = 30, *F* = 30) children with a mean age of 11.2 years (SD = 1.89; range 7–15 years) were recruited from a trampoline summer camp in ON, Canada. Permission to approach the children was granted by the head coach of the trampoline club. Informed consent from the parents of participants and assent forms from the participants were obtained prior to any participation. The experiment was approved by the ethics review board at the University of Ottawa in order to ensure that all ethical standards were met.

### Materials and task

The to-be-learned task involved skill sequences performed on a double mini-trampoline apparatus^1^. The task goal given to the participants was to advance as far as possible through a progression sequence of pre-determined double mini-trampoline skills. Two individuals were involved in determining (a) the progression sequence used such that the skills progressed from the easiest double mini-trampoline skill sequence to skill sequences of greater difficulty, and (b) specific criteria for each progression sequence that determined whether the jump sequence was performed correctly. These two individuals were experienced in double mini-trampoline with combined experiences of being competitors (10 years), coaches (18 years), and/or judges (18 years) of the sport.

A Sony video Handycam (model number DCR-HC65/HC85) mounted on a tripod was used for two purposes; one involved creating a skilled model video of progression sequences used in the to-be-learned task and the second was to videotape the children throughout the experiment. For both, the video camera was set up on a tripod at a 45° angle to the double mini-trampoline, capturing the side-frontal view. This angle of viewing was used because it provided the best angle for learners to observe critical components of the progression.

A Toshiba laptop computer was used to show a skilled model video to the participants prior to commencement of the acquisition phase as well as the video footage of the participant’s previous performance during acquisition. The Dartfish software (version 4.5.1.0) was used to enable the last trial to be seen on the laptop after a pre-determined delay period of 5 s. The skilled model video that was created consisted of four of the progression sequences performed by a skilled model who was an adult female and a former national level double mini-trampoline competitor.

### Measures

#### Physical performance

Each level of the progression sequence had certain criteria that were to be obtained before the participant could progress to the next level. Ten of the criteria were consistent for each progression sequence and then each progression sequence also had criteria specific to that jump sequence (see Table 1). For acquisition, physical performance scores were based on the progression level attained at the end of each trial block. Physical performance scores for retention were the number of progression levels advanced during the retention test, plus the percentage value of the number of criteria attained at the current progression for the remaining trials. For example, if a participant started at level 6 and advanced to level 8 by trial 5, but did not advance to level 9, the trials at level 8 would be evaluated and the correct number of criteria per trial would be calculated. To continue with the example, if trials 5 and 6 showed that the participant could do 7 of the 14 criteria in that sequence on both trials, the retention score would be 2.50.

**Table 1:** **Standard criteria for progressions 1–17**.

Standard criteria^a^
1. Push off the runway with dominant foot
2. Two foot landing onto the first mini
3. Land in the white area on the first mini
4. Two foot landing onto the second mini
5. Land in the white area on the second mini
6. Arms move up to ears when in the air on first skill
7. Arms move up to ears when in the air on second skill
8. Two foot landing
9. Proper landing (3 s control)
10. Land in the box on the mat

*^a^Other criteria were used that were specific to each jump sequence that are not listed here*.

Physical performance scores were evaluated by a former national level competitive double mini trampolinist (primary evaluator). Prior to scoring all performances, a research assistant (with a background in gymnastics) was trained by the primary evaluator with the scoring criteria related to each progression sequence. Both the primary evaluator and the research assistant, blind to the experimental condition of the participant on video, scored 20% of the videos to determine inter-rater reliability of the scoring criteria. A correlation between the two judges’ scores was calculated and revealed strong reliability levels; *r* = 0.93.

#### Self-efficacy

The self-efficacy measure was developed according to Bandura’s (2006) guidelines. The instructions explained that the participants were to evaluate their beliefs in their ability to perform the stated double mini-trampoline jump sequence while meeting all of the necessary criteria needed to advance to the next progression. Seven statements progressed from easier skill sequence progressions to more difficult progressions. At the top of the questionnaire, just after the instructions, was a Likert scale from 1 to 100, in increments of 10, with the anchors as “*cannot do at all*” and “*highly certain can do*,” and “*moderately can do*” was placed under the 50 value of the scale. An example of an item is “I can get tuck jump to tuck jump.” An average score of all the items was calculated to represent participants’ perceived self-efficacy. Cronbach alpha values were considered good at all three time points (αs = 0.942–0.949).

#### Intrinsic interest and perceived choice

Two subscales from the Intrinsic Motivation Inventory (IMI), which has been tested for validity and reliability by McAuley et al. (1987) were used; specifically, the interest/enjoyment and perceived choice scales. Each scale has individually displayed satisfactory validity and reliability (McAuley et al., 1987). Past research that has used the IMI has revealed that the subscales can be used separately from one another without affecting the validity of the results (Ryan et al., 1991). As such, one can choose subscales that are relevant to the issues being explored. For both subscales, a Likert scale ranging from 1 to 7 is used to indicate whether each statement is *not at all true* (1) or *very much true* (7). The interest/enjoyment was chosen because, although the measure is called the IMI, this is the only particular subscale that assesses intrinsic motivation directly. This subscale consists of seven items, modifiable to the specific task. An example of an item is “I enjoyed doing the double mini activities very much.” The perceived choice scale was included to provide a measure of autonomy. This subscale also consists of seven items and an example of an item is “I believe I had a choice about doing the double mini activities.” Perceived choice and interest enjoyment scores were calculated separately. Average subscale scores were used, thus giving a number for each participant ranging from 1 to 7. For the current data set, Cronbach alpha values were good at all three time points for the intrinsic interest scale (αs = 0.838–0.918); however they were in the weak to acceptable range for the perceived choice scale (αs = 0.634–0.732), and should be interpreted with caution.

#### Perceived success

A one item questionnaire that had the statement “How successful were you on that last trial?” was used to measure perceived success on those trials in which KP was provided. A Likert scale from 1 (*unsuccessful*) to 7 (*very successful*) was provided to the participants. The average perceived success per block was calculated for each participant, thus resulting in a number ranging from 1 to 7.

### Procedure

**Figure 1:**
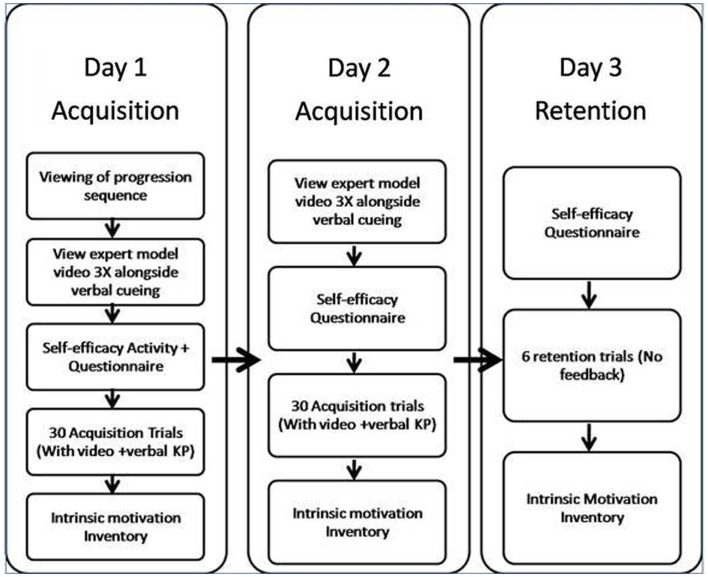
**Summary of procedure during the acquisition (day 1–2) and retention (day 3) phases**.

The experiment was completed over 3 days, and involved two phases; acquisition and retention. Participants were typically tested in groups of 3–4 (range was 2–7 people). A cluster-randomized design was used such that recruitment week acted as the cluster. The recruitment started with the LC condition because we needed to have the participants’ self-observation frequency pattern in order to prescribe the schedule for those that were assigned to the EC group (yoked group). See Figure 1 for an overview of the experimental protocol.

#### Acquisition phase

The acquisition phase consisted of two testing days; both of which involved the completion of 30 practice trials (5 blocks of 6 trials). On day 1, before beginning any trials, participants were first shown a list of the 17 trampoline progressions and the necessary criteria that were to be accomplished in order to move from one progression to the next. After having read the progression sheet, they watched a video of a skilled model performing the first and third level of the double mini-trampoline progressions. This video was viewed three times consecutively. While viewing the video, a researcher verbally cued the specific criteria for the participants to focus on during each trial to ensure that the participants understood the criteria needed to advance from one progression to the next. All of the necessary criteria within the modeled progressions were covered verbally within those three viewings. The researcher explained to the participants that most of the criteria were consistent from one progression to the next, and when new criteria were needed, they would be explained.

Following the viewing of the skilled model video, the self-efficacy measure was administered. Bandura (2006) recommended practice trials with another task in order to ensure participants understand the concept of self-efficacy. Thus, we had the children first perform a jumping task. The participants lined up on the short end of a mat which was made up of colored stripes, approximately 2 ft in width each. Prior to each jump they were asked to estimate on a scale of 1–100 their belief in their ability execute the jump to be attempted, with each jump distance increasing. This provided them with a concrete analogy to understand the self-efficacy questionnaire used for the double mini-trampoline progressions. Following this jumping exercise, participants were asked to complete the self-efficacy questionnaire specific to seven of the double mini-trampoline jump sequences where they rated their degree of confidence by circling a number from 0 to 100.

Subsequent to the self-efficacy questionnaire, the participants were given 30 practice trials, with all participants beginning at progression level one. They were informed that the goal was to advance as far through the progressions as they could by the end of acquisition. In the occurrence that a participant moved onto a progression beyond the ones shown by the skilled model video that day, the researcher verbally instructed which new criteria were essential.

All participants received self-observation that included attentional cueing and transitional statements; however, the control of viewing the self-observation video differed between the LC and EC group. The LC group had full control over when they watched the self-observation video. The laptop replayed their last trial with a 5-s delay, which offered them enough time to finish their jump sequence and walk over to the laptop. In contrast, the EC group had no control over their schedule; their self-observation schedule depended solely on when their age and gender matched counterpart in the LC condition had chosen to view the video. The experimenter told the participant after the skill sequence to watch the video.

Although the LC and EC groups differed in respect to controlling when they viewed the self-observation video, all other procedural elements were the same. First, prior to viewing the video feedback, participants were asked to rate their perceived success on their last trial by responding to the one item questionnaire and pointing to the number on the Likert scale that best represented their perceptions. After recording the participant’s response, an instructor verbally cued the participant as to where a critical inaccuracy existed in the movement, based on a pre-established priority list. For example, the instructor may have told the participant to “focus on where your arms are during your first skill.” Following the videotape viewing, the instructor verbalized a transitional statement which emphasized how the participant could address the performance inaccuracy within his/her next trial. In follow up to the previous example, the instructor would state “on your next trial, bring your arms up to your ears when pushing off the trampoline.” Following the completion of the 30 acquisition trials, the participants completed the chosen subscales of the IMI. They were instructed to rate the truthfulness of each statement on the seven-point Likert scale by circling the number most representative of their perceptions.

The second day of acquisition followed the same protocol as the first, with the following exceptions: (a) the progression sheet was not reviewed, (b) the skilled model video showed two higher levels of the progression sequence and the accompanying criteria were provided, (c) the children did not perform the distance jumping exercise prior to administration of the self-efficacy questionnaire, and (d) participants began this set of acquisition trials at the progression level they had reached the previous day.

#### Retention

The retention phase took place 24 h after the beginning of the second acquisition day. The participants began by completing the self-efficacy questionnaire, and then each participant physically performed one block of six trials. Participants began at a progression level that was two levels lower than the highest level attained the previous day. They were encouraged to advance as far as they could through the progressions within the six retention trials. Similar to the acquisition, the learners would only advance to the higher progression level when all of the required criteria were met in the jump sequence. No video feedback was provided within this phase. Once the block of six trials was completed, the participants completed the chosen subscales of the IMI a final time.

## Results

### Preliminary analyses

Based on stem and leaf-plot analysis, four participants were identified as outliers with respect to the psychological variables and their data was not included in further analysis of the self-efficacy, intrinsic motivation, and perceived choice data, resulting in a final sample size of 56 for those analyses. All 60 participants’ data was included in the performance and perceived success analyses.

### Acquisition

A two-way MANOVA confirmed that the LC and EC groups were not significantly different with respect to their perceptions of self-efficacy, intrinsic interest, and perceived choice on day 1 (*p*s > 0.05). Group means and standard deviations for acquisition and retention are shown in Table 2. A separate independent samples *t*-test confirmed that the two groups did not differ significantly in their performance following block 1 (*p* > 0.05). This suggests that both groups were similar with respect to all variables of interest on day 1 of the acquisition phase.

**Table 2:** **Means (standard deviations) for self-efficacy, intrinsic interest, and perceived choice at each time point by group**.

	Acquisition day 1		Acquisition day 2		Retention	
	LC	EC	LC	EC	LC	EC
Self-efficacy	69.4 (18.76)^a^	76.95 (12.96)	75.74 (15.47)^a^	74.21 (15.59)	80.13 (16.81)	79.55 (12.36)
Intrinsic interest	6.4 (0.60)	6.02 (1.10)	6.53 (0.58)	6.13 (0.90)	6.69 (0.45)^b^	6.16 (0.88)^b^
Perceived choice	6.44 (0.58)	6.1 (0.83)	6.57 (0.54)	6.17 (0.84)	6.7 (0.52)^c^	6.13 (0.83)^c^

*Self-efficacy was assessed using a 1–100 scale while intrinsic interest and perceived choice were assessed using a 1–7 scale. LC, learner-controlled group; EC, experimenter-controlled group*.

*^a, b, c^Values with the same superscript of letters a, b, or c denote significant differences at *p* < 0.05*.

**Figure 2:**
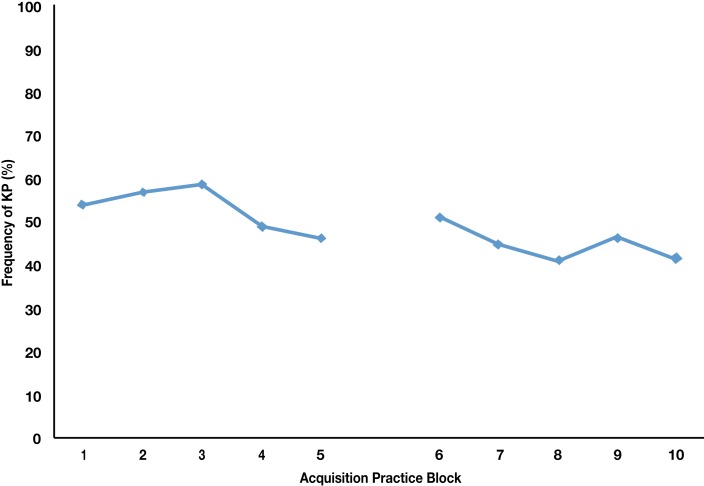
**Frequency of knowledge of performance (KP) requested by participants in the learner-controlled group across days 1 and 2 of the acquisition phase**.

To examine the nature of the feedback schedule requested by the LC group, a one-way ANOVA with repeated measures was performed on the frequency of self-observation requested by participants across each of the 10 acquisition blocks. The frequency score is expressed as a percentage and was calculated for each block of trials based on the number of KP requests per number of trials. Results demonstrated a significant main effect for time, *F*(9, 252) = 2.29, *p* < 0.05, $\eta_{p}^{2}=0.076,$ observed power = 0.90, with the number of requests decreasing across the acquisition period. Pairwise comparisons revealed a significant difference, *t*(29) = 3.62, *p* < 0.05, between the amount of feedback requested in block 2 (*M* = 56.9; SD = 23.8) and block 8 (*M* = 40.8, SD = 25.8); all other pairwise comparisons were non-significant (*p*s > 0.05). The main effect for time and direction of the means suggest that the LC group followed a fading feedback schedule (Figure 2).

A 2 (Group: LC, EC) × 2 (Time: day 1, day 2) MANOVA with repeated measures for time was used to examine differences between the LC and EC groups in terms of self-efficacy, intrinsic motivation, and perceived choice across acquisition. The analysis revealed a group by time interaction for self-efficacy, *F*(1, 54) = 4.89, *p* < 0.05, $\eta_{p}^{2}=0.083,$ observed power = 0.58. Separate one-way ANOVAs with repeated measures for time were performed separately for each group to examine changes in self-efficacy. Results revealed that the LC group’s self-efficacy scores increased across acquisition at a level approaching significance, *F*(1, 26) = 4.04, *p* = 0.055, $\eta_{p}^{2}=0.134,$ observed power = 0.49, while the EC group’s did not change significantly (*p* > 0.05). There were no main effects or interactions with respect to intrinsic interest or perceived choice during acquisition (*p*s > 0.05).

**Figure 3:**
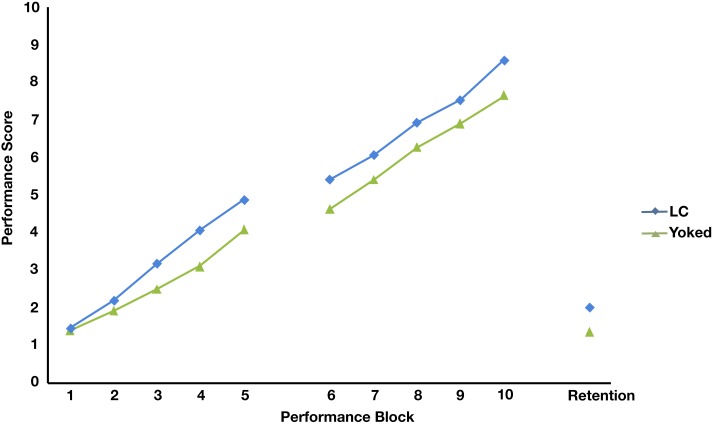
**Performance scores of participants in the learner-controlled (CL) and Yoked groups across all three experimental phases**. Performance scores from block 1–10 reflect the progression level reached at the end of that trail block. Performance scores in retention reflect the sum of the number of progression levels advanced during retention and percentage of criteria attained towards the next progression level.

A 2 (Group) × 10 (Trial Block) ANOVA with repeated measures for time was calculated to examine differences between the two groups’ performance across acquisition. There was a main effect for time, *F*(9, 522) = 176.72, *p* < 0.05, $\eta_{p}^{2}=0.753,$ observed power = 1, with pairwise comparisons showing performance scores increasing significantly across all blocks (*p*s < 0.05). As seen in Figure 3, both groups progressed through the double mini-tramp progressions in a linear fashion during both acquisition days. In some blocks of trials, KP was not provided and thus perceived success scores were not available for every acquisition block. Consequently, an independent samples *t*-test was used to examine differences in perceived success. This analysis revealed that LC self-observation had a moderate effect on mean perceived success ratings in acquisition, *t*(58) = 2.37, *p* < 0.05, *d* = 0.542. LC group participants reported significantly higher perceived success (*M* = 5.46, SD = 0.81) compared to the EC group (*M* = 4.99, SD = 0.69).

### Retention

Table 2 shows the descriptive statistics for the three questionnaires used in retention. A two-way MANOVA on the retention data demonstrated that the LC group scored significantly higher than the EC group in intrinsic motivation, *F*(1, 54) = 7.63, *p* < 0.05,$\eta_{p}^{2}=0.124,$ observed power = 0.77, and perceived choice, *F*(1, 54) = 9.40, *p* < 0.05, $\eta_{p}^{2}=0.148,$ observed power = 0.85. No significant differences in self-efficacy (*p* > 0.05) between the two groups, however, were obtained. A separate independent samples *t*-test showed that the LC group also had significantly higher performance scores compared to the EC group at retention, *t*(58) = 3.21, *p* < 0.05, *d* = 0.753.

A hierarchical multiple regression analysis was performed to explore whether the intervention (i.e., group condition) and psychological variables predicted performance at retention. Group (dummy coded as LC = 1, EC = 0) was entered on the first step, with the psychological variable scores from the retention test (self-efficacy, intrinsic motivation, perceived choice) entered on the second step. The inclusion of both group and psychological variables predicted performance at retention, *F*(4, 55) = 4.99, *p* < 0.05, $R_{\text{adj}}^{2}=0.225.$ The only significant predictors, however, were group, β = 0.489, *p* < 0.05, and self-efficacy,β = 0.313, *p* < 0.05.

## Discussion

The aims of this research were twofold. First we were interested in determining whether LC KP obtained through self-observation on video provided a learning advantage for children. Second, we sought to examine whether the possible learning advantages could be explained within a self-regulation framework, with a specific interest on differences in self-efficacy and intrinsic motivation. These self-regulatory processes fall within the self-motivational beliefs component of the forethought phase of Zimmerman’s (2000) model of self-regulated learning; the phase that involves those beliefs and processes that occur prior to the initiation of a given event. We also relied on previous results that showed that children in LC environments for KR chose to receive feedback after good trials (Chiviacowsky et al., 2008a) to set our hypotheses. In particular, we expected that the children would choose to self-observe after successful trials. A logical extension of this is that viewing successful trials could build self-efficacy for progressing through the jump sequences; consequently, it was expected that higher levels of self-efficacy would be obtained for the LC as opposed to the experimental-controlled group. Moreover, the LC group was also expected to show greater intrinsic motivation and perceptions of autonomy than that of the EC group.

Given the importance of the concept of relative permanence for interpreting learning effects (Schmidt and Bjork, 1992), our interest laid mainly with the results in of the retention phase. In terms of our first research question, we did see LC benefits for children who were able to choose when they wanted to self-observe as opposed to those who were provided the same self-observation schedule, but under an EC setting. Children in the LC group were able to progress further in the double mini-trampoline skills during retention than the EC group. These findings further reinforce the growing literature on the effectiveness of LC environments for motor skill acquisition and extend the reach to include the benefits associated with the control of KP by children.

These learning advantages for LC self-observation are similar to those found by Janelle et al. (1997) with adults. Unlike Janelle et al.’s results, however, our sample of children requested the KP information for a larger percentage of trials (close to 30%) than that demanded by their adult population (11.5%). Chiviacowsky et al.’s (2008a) results also showed children requesting KR at a similar frequency to that obtained here. One potential explanation for the apparent differences between adults and children in feedback frequency relates to self-evaluative processing. That is, it is possible that the children were asking for KP information for a high percentage of trials, due to the greater need for them to compare the intrinsic feedback received from execution of the task with extrinsic sources of information. Notable, though, is that there was still a fading pattern apparent in the feedback schedule; highlighting that children were likely transferring the common criteria across the progression sequences and developing an error detection and correction mechanism. Regardless, these higher requested frequencies reinforce the idea that we must consider differences in information processing between adults in children; such as speed of processing and working memory (Surwillo, 1977; Thomas, 1983), when implementing teaching strategies for motor skill learning for children.

Important to consider, however, is that the task used in this experiment differed from that used by Janelle et al. (1997) as their participants were continually trying to improve on the same throwing task across all trials. In our situation, the task was constantly changing because once the learner had mastered one progression level, they immediately moved onto a higher level which would require different movement patterning in the jump sequence. Consequently, it may be that that KP information was used at a greater frequency here for the children as others have shown that learners benefit from a higher frequency of feedback when learning difficult tasks (e.g., Swinnen et al., 1997; Wulf et al., 1998). As such, further experimentation should attempt to tease out possible differences in LC scheduling between children and adults while also controlling for task characteristics.

Knowing that children do gain learning advantages from a LC environment obviously leads one to ask why these benefits emerge; thus identifying the second objective of our research. In this regard, we worked within Zimmerman’s self-regulation of learning framework and were influenced by previous results that showed participants preferred to obtain KR following perceived good trials as compared to poor trial performances (Chiviacowsky et al., 2008a). Indeed, our analyses showed that participants in the LC group had higher levels of perceived success than those in the EC group. Further, the LC group showed greater positive change in their self-efficacy during acquisition as compared to their yoked counterparts. Thus, our reasoning that requesting KP after “good” trials would likely increase self-efficacy seems substantiated. It may be that the participants’ self-evaluation of their performance (perceived success) and actual performance (self-observation video) were congruent, leading to the noted elevations in self-efficacy. Anecdotally, many of the participants would state that they did not want to view a particular trial because they knew they had executed specific criteria incorrectly and did not want to see their performance, suggesting self-evaluations of the performance had already occurred. We, however, did not gain full information on these self-evaluative processes and it would be insightful if future research examined them in more detail in relation to self-control. No differences, however, were obtained in the retention phase for self-efficacy, and thus, the permanence of these self-efficacy effects is questionable.

Despite no significant differences for self-efficacy in retention, the retention results showed that LC environments for KP had significant positive effects on intrinsic motivation, and perceptions of autonomy. These combined results suggest that, early in learning, a LC environment may be important for developing learners’ self-efficacy and forming expectations for performance, while later, it may contribute to greater feelings of intrinsic motivation and autonomy that subsequently enhances motor skill learning. No explicit measures concerning the greater perception of control specific to the scheduling of the KP, however, were obtained and we recognize this as a limitation of our design. We recommend that future research on this topic examine more directly such perceptions of control over the variable of interest.

Another limitation concerning our interpretation, however, is that we used analyses of variance, which do not allow us to examine how the variables interact with performance across time in acquisition and retention. A preliminary examination into whether self-efficacy, intrinsic motivation, and perceived autonomy could explain the benefits associated with LC versus EC environments was conducted via a multiple hierarchical regression with the retention data. While this analysis suggested that receiving the LC intervention had the largest effect on performance scores at retention, intrinsic motivation and perceived choice were not significant predictors despite the group differences attained in the MANOVA. Self-efficacy perceptions during retention, however, were a significant predictor of physical performance benefits. This was surprising given this measure showed significant differences during acquisition only and not during retention. These findings highlight the complexity of understanding the mechanisms related to the benefits of LC environments. Future research needs to be conducted which enables a more thorough exploration into the interactions and time effects associated with LC outcomes.

In conclusion, our results showed that LC environments enhanced the learning of double mini-trampoline skills. Simultaneously, greater changes in self-efficacy during acquisition occurred for the LC group, as compared to the EC group, yet no group differences were found during retention. Greater intrinsic motivation and perceived choice during retention, however, were evidenced for the LC group as opposed to the EC group. In spite of these differences in intrinsic motivation and perceived choice at retention, hierarchical regression analysis only had group assignment and self-efficacy significantly predicting physical performance scores. These results suggest that further investigation is required before making definitive conclusions about the underlying mechanisms of learner-controlled advantages in motor skill acquisition.

## Conflict of Interest Statement

The authors declare that the research was conducted in the absence of any commercial or financial relationships that could be construed as a potential conflict of interest.

## Footnotes

^1^Double mini-trampoline skills are accomplished by running down a 15-m runway and jumping onto an inclined small trampoline bed that allows one to take flight and perform a skill (e.g., a star jump). A second flat trampoline bed of the same size is connected to the first trampoline bed and located a short distance away. Double mini involves two types of passes. The first pass (i.e., mount pass) entails performing the first aerial skill from the inclined trampoline bed to the flat one, and a second aerial skill is immediately performed (e.g., a pike jump) from the flat trampoline bed onto a cushioned landing mat. The second type of pass (i.e., spotter pass) involves doing the first aerial skill from the inclined trampoline bed to the flat bed, performing and landing a second aerial skill on the flat bed, and then performing a third aerial skill onto the landing mat. Thus, each skill sequence involved connecting aerial skills on the trampoline beds that finish with a controlled landing.
